# Influence of micronutrients on anxiety and depression in patients with amyotrophic lateral sclerosis

**DOI:** 10.3389/fnut.2026.1790327

**Published:** 2026-07-13

**Authors:** Sandra Sancho-Castillo, Guillermo Bargues-Navarro, Ruth Gabriela Cárdenas Salazar, Jesús Privado, Elena Sanchis-Sanchis, David Sancho-Cantus, Claudia Emmanuela Sanchis-Sanchis, Laura Cubero-Plazas, Mónica Cano Mateu, José Enrique de la Rubia Ortí

**Affiliations:** 1 Department of Pathology, Catholic University of Valencia San Vicente Mártir, Valencia, Spain; 2 Doctoral School, Catholic University of Valencia San Vicente Mártir, Valencia, Spain; 3 Department of Medicine and Health Sciences, Catholic University of Valencia San Vicente Mártir, Valencia, Spain; 4 Department of Psychobiology and Methodology of Behavioral Sciences, Complutense University of Madrid, Madrid, Spain; 5 Department of Nursing, Catholic University San Vicente Mártir, Valencia, Spain; 6 Department of Basic Biomedical Sciences, Catholic University of Valencia San Vicente Mártir, Valencia, Spain

**Keywords:** amyotrophic lateral sclerosis, anxiety, depression, micronutrients, neurodegenerative disease

## Abstract

**Background:**

Amyotrophic lateral sclerosis (ALS) is a neurodegenerative disease characterized by a progressive loss of motor function, with respiratory failure representing the leading cause of death. Beyond motor deterioration, ALS involves extramotor manifestations, including prominent anxiety and depression symptoms. The intake of certain micronutrients has been associated with these emotional symptoms, due to their role in monoaminergic and peptidic neurotransmitter synthesis pathways; furthermore, these emotional symptoms can severely reduce patients’ quality of life. Therefore, this study aims to analyze the relationship between the intake of key micronutrients related to neurotransmitter activity and anxiety and depression symptoms in ALS patients.

**Methods:**

A cross-sectional study was conducted with 61 patients with bulbar- or spinal-onset ALS. Dietary micronutrient intake (iodine, magnesium, vitamin B6, vitamin B9, and vitamin C) was assessed using a seven-day dietary record and a Food Frequency Questionnaire. Depression was assessed using the ALS Depression Inventory-12 (ADI-12), while anxiety-related symptoms were assessed using the Beck Anxiety Inventory (BAI).

**Results:**

A path analysis was conducted to examine the statistical prediction of emotional measures from nutrient intake. The model showed a good fit to the data: NFI = 1.000, GFI = 1.000, and SRMR = 0.001. Considering the magnitude of the effect of the regression weights (*β* ≥ 0.14), depression scores exhibited exploratory associations with iodine (*β* = −0.21) and magnesium (*β* = 0.22), while vitamin B9 (*β* = −0.32) emerged as an inferentially significant predictor (*p* < 0.05). Anxiety-related symptom severity (BAI scores) showed exploratory associations with iodine (*β* = −0.31), vitamin B6 (*β* = 0.24), and vitamin C (*β* = −0.15), whereas depression scores (*β* = 0.51) were an inferentially significant predictor (*p* < 0.05), with 41% of explained variance.

**Conclusion:**

In this sample, lower vitamin B9 intake was statistically associated with depression scores in patients with ALS, while lower iodine and higher magnesium intake showed exploratory associations. Regarding anxiety-related symptoms assessed using the Beck Anxiety Inventory (BAI), higher scores were statistically associated with lower iodine and vitamin C intake, higher vitamin B6 intake, and elevated depression scores, and showed exploratory associations with lower iodine and vitamin C intake, and higher vitamin B6 intake.

## 1 Introduction

Amyotrophic lateral sclerosis (ALS) is among the most devastating neurodegenerative pathologies due to its rapid progression and fatal outcome. In Spain, the annual incidence is 1.4 per 100,000 inhabitants (1), showing a higher prevalence in males and a peak at 65 years of age (2). The disease presents two primary clinical forms of onset: bulbar (25%, characterized by dysarthria and dysphagia) and spinal (70%, manifesting as weakness in the upper or lower limbs) (3). Its etiology combines advanced age, genetic and environmental factors (such as smoking, alcoholism and traumatic injuries). Consequently, cases are classified as either familial ALS (5–10%) or sporadic ALS (90%) (4, 5).

ALS primarily affects the nerve cells responsible for muscle control, causing weakness, atrophy, dysphagia, dysarthria, and dyspnea. Among these, with respiratory failure remains the leading cause of death, typically occurring within 3 years of diagnosis (6). However, in addition to motor deterioration, ALS causes extramotor symptoms. Notably, anxiety and depression frequently affect both patients and caregivers (7–9).

Anxiety symptomatology is one of the most frequent psychiatric comorbidities in ALS. Approximately 18–26% of patients present clinically significant anxiety; although other authors indicate that 33% show anxiety symptoms (10), Furthermore, some longitudinal studies raise the percentage to 60% following the initial diagnosis and during disease progression (11). These symptoms substantially decrease quality of life and increase mortality risk. Anxiety-related clinical manifestations include fatigue, fear, nightmares, loss of interest, memory alterations, bruxism, spasms, tachycardia, palpitations, tinnitus, dyspnea, and choking sensation (12).

Depression, another prevalent comorbidity, manifests as persistent sadness, irritability, a feeling of emptiness, and anhedonia (ICD-11) further compromising the patient’s quality of life. According to DSM-IV criteria, approximately 7% of patients diagnosed with ALS present mild depression, while 5% meet the criteria for major depressive disorder (DSM-IV criteria) (13). However, global consensus regarding these percentages is still lacking due to substantial cross-country variations. For example, prevalence rates range between 10 and 40%in Brazil, whereas 15.38% of patients presented depressive syndrome in Portugal (10, 14).

Ultimately, both anxiety and depression symptoms exert a profound impact on disease progression and quality of life (15). From a physiological standpoint, certain micronutrients play critical functions in mental health. They act as enzymatic cofactors, antioxidants, and modulators of gene expression that determine the synthesis, release, and signaling of neurotransmitters. In this regard, vitamin C, magnesium, and selenium are hypothesized to exert protective effects against the risk of developing anxiety symptoms, probably due to their role in modulating central neurotransmitter systems (16). Specifically, vitamin C directly influences oxytocin synthesis by facilitating the reactions of the peptidyl glycine alpha-amidating monooxygenase (PAM), enzyme which catalyzes the activation of various hormones and neurotransmitters (17). Similarly, folates (vitamin B9), through their participation in the methionine cycle and tetrahydrobiopterin regeneration, also modulate serotonin and dopamine synthesis. Concurrently, vitamin B6 acts as an essential cofactor for L-amino acid decarboxylase, the rate-limiting enzyme involved in the conversion of precursors to serotonin and dopamine (18, 19). Iodine, in turn, indirectly influences monoaminergic neurotransmission through thyroid hormones, which regulate receptor expression in limbic brain regions (20). Furthermore, magnesium and zinc also participate in serotonin synthesis and modulate glutamatergic transmission, while iron acts as a cofactor for tryptophan hydroxylase and tyrosine hydroxylase enzymes, required for serotonin and catecholamine synthesis, respectively (21).

The biochemical pathways of these micronutrients, which are based on their direct participation in monoaminergic and peptidic neurotransmitter synthesis, provide a plausible framework for understanding how nutrient variations might correlate with neuronal receptor sensitivity. Understanding these mechanisms establishes the theoretical foundation for evaluating how specific micronutrient intake is statistically associated with, or acts as a potential predictor of, anxiety and depression scores.

Therefore, the main objective of this study is to analyze the statistical relationship between the intake of key micronutrients related to neurotransmitter activity and the corresponding scores of anxiety-related and depression symptoms in patients with ALS.

## 2 Methodology

### 2.1 Study design

This is an analytical, clinical, observational, cross-sectional, and quantitative study evaluating patients diagnosed with bulbar- or spinal-onset ALS.

### 2.2 Participants

Patients with bulbar or spinal ALS from different regions of Spain were recruited through ALS associations affiliated with FUNDELA. After being informed about the nature of the study and expressing interest in participating, all participants provided written informed consent. The applied inclusion criteria were men aged ≥18 years; women aged 18–50 years; women aged >50 years who were non-fertile; a confirmed ALS diagnosis for ≥6 months; and ongoing treatment with riluzole. Conversely, the exclusion criteria were tracheostomy; invasive or non-invasive positive pressure ventilation; participation in another clinical trial within the previous 28 days; cognitive impairment due to dementia; harmful alcohol or drug consumption; hepatitis B or C; human immunodeficiency virus (HIV); renal insufficiency (doubled serum creatinine); and liver dysfunction (alanine aminotransferase (ALT) or aspartate aminotransferase (AST) levels three times higher than normal).

After applying these criteria, the final sample consisted of 61 ALS patients with a mean age of 56.43 years (*SD* = 10.17 years). Of these participants, 65.60% were male, 16.40% presented with bulbar-type ALS and 83.60% presented with spinal-onset type. The mean time since diagnosis was 28.29 months (*SD* = 29.43 months).

### 2.3 Measures and instruments

To assess depression levels, the ALS Depression Inventory-12 (ADI-12) was used. This instrument consists of 12 items designed to evaluate depressive symptoms specifically in individuals diagnosed with ALS. The evaluation focuses on the patient’s mood experienced during the 2 weeks prior to the interview and uses a four-point Likert scale, ranging from “Strongly agree” to “Strongly disagree” (22). The internal consistency (Cronbach’s alpha) of the inventory in our sample was 0.920, indicating excellent reliability (23).

To assess anxiety-related symptom severity, the BAI was used. This instrument consists of 21 items designed to evaluate the intensity of anxiety symptoms through self-report. Each item corresponds to a specific anxiety symptom, and participants indicate the degree to which they experienced it during the past week, using a four-point Likert scale ranging from “Not at all” to “Severely—I could barely stand it” (24, 25). In our sample, Cronbach’s alpha was 0.909 for the somatic scale, 0.922 for the affective-cognitive scale, and 0.952 for the total scale.

Dietary assessment protocol and instrument integration: Micronutrient intake was assessed using seven non-consecutive 24-h dietary recalls, complemented by a semi-quantitative Food Frequency Questionnaire (FFQ), which together provided a comprehensive characterization of participants’ habitual dietary patterns (26). All dietary information was self-reported and subsequently reviewed in an individual interview with a registered nutritionist, who examined both instruments for completeness and internal consistency and clarified any missing or ambiguous entries.

Nutrient intakes from the 24-h recalls were calculated using DietoPro®,^1^ which is based on the Spanish Food Composition Database (BEDCA). For each participant, daily nutrient intakes from the seven recalls were averaged to obtain a single estimate of usual intake. Total energy intake was calculated for each recall, and nutrients were expressed as absolute values; energy-adjusted intakes were derived using the residual method in secondary analyses.

Implausible energy intakes were identified using predefined cut-offs and examined individually. Extreme nutrient values were inspected for potential reporting inconsistencies; when no error could be identified, they were retained in the main analyses but evaluated in sensitivity analyses excluding outliers. Given the unexpectedly high variability observed for vitamin B6, individual records contributing extreme values were examined in detail, and sensitivity analyses excluding these values were performed to ensure the robustness of the nutrient estimates.

Nutritional supplementation was assessed in each 24-h recall and during the nutritionist interview. All participants confirmed that they had not taken vitamin or mineral supplements for at least 1 month before the assessment, ensuring that nutrient intake estimates reflected habitual diet alone.

In each 24-h recall, participants documented all foods and ingredients consumed, using standardized household measures or exact weights with guidance from an equivalence chart. The FFQ (27) assessed the long-term frequency of consumption for each food group and was used to characterize dietary patterns and to check the consistency of reported intakes.

### 2.4 Data analysis

First, the distribution of nutritional and emotional measures (anxiety-related and depression symptoms) was analyzed to assess normality. Second, Spearman rank correlations were calculated between these emotional and nutritional measures to detect association patterns between both types of variables. Third, a structural equation modeling model (path analysis) was estimated to evaluate which nutrients statistically account for the scores of the two emotional symptoms (anxiety-related and depression symptoms). The first two analyses were performed using the SPSS v. 23 statistical package and the third using AMOS v. 23 (28).

To assess the fit of the data to the path model, two types of goodness-of-fit indices were used: absolute fit indices, which evaluate whether the theoretical model fits the empirical data: the *χ^2^/df* ratio (29), where values less than 3 typically represent an acceptable mathematical threshold; the Goodness-of-Fit Index (GFI) (30), with values greater than 0.95 representing a good fit; and the Standardized Root Mean Square (SRMR) (31), with values less than 0.08 indicating a good fit (23); additionally, the presence of less than 5% of standardized residuals with an absolute value greater than 2.58 was considered a good fit criterion (32); and incremental fit indices, which compare the obtained model with the null model, including the Normed Fit Index (NFI) (29), with values greater than 0.95 indicating a good fit. A sample of between 5 and 10 participants per variable is recommended for this type of model (23, 32). In our case, the sample included 61 participants and 7 variables (with a ratio of 61/7 = 8.71 ≈ 9), which indicates an adequate sample-to-variable ratio. When working with small samples, it is recommended to use the bootstrapping parameter estimation technique (32). This technique creates replicates of the original sample of equal size by drawing with replacement, thereby estimating the population parameters and allowing for an analysis of the representativeness of sample statistics in the target population. In our case, we estimated 500 bootstrap samples of size *n* = 61 from the model under test. In addition, a *post-hoc* statistical power (1- *β*) analysis was also conducted to verify whether the sample size was adequate for the tested model, based on the obtained *R^2^* values, the number of predictors, and a significance level of 0.05. The recommendation is that statistical power should reach a minimum value of 0.80 (23) for the results to be considered statistically robust.

## 3 Results

### 3.1 Descriptive results

Table 1 presents the descriptive statistics of both nutrient intakes associated with neurotransmitter activity and scores of anxiety-related and depression symptoms of the studied sample. Variables with skewness value no greater than | ± 2.00| and a kurtosis value no greater than | ± 7.00| were considered to follow a normal distribution (33).

**Table 1:** Descriptive statistics of nutrients and emotional measures.

Measures	*M*	*SD*	Asymmetry	Kurtosis
Iodine (μg)	218.60	246.08	3.53	13.69
Magnesium (mg)	398.41	223.90	1.33	2.50
Vitamin B6 (mg)	14.26	64.30	5.41	28.31
Vitamin B9 (μg)	318.03	157.51	1.37	3.25
Vitamin C (mg)	174.55	114.23	1.42	2.81
Depression (ADI-12)	10.03	7.58	1.22	1.58
Anxiety (BAI)	35.98	13.37	1.05	0.41

### 3.2 Correlations

Because some of the measures were not normally distributed, the Spearman rank correlation between the variables was calculated. Table 2 displays the Spearman correlations between nutrient intake and the scores of anxiety-related and depression symptoms. According to Cohen’s criteria (34), correlation coefficients of 0.10 represent a small effect size, whereas values of 0.30 and 0.50 indicate medium and large effect sizes. Statistically significant correlations were defined as those with values of at least | ± 0.25| at the 5% significance level. Considering this criterion, vitamin B9 emerged as the statistically relevant micronutrient for depression scores, whereas all evaluated micronutrients with the exception of vitamin B6 showed significant correlations with anxiety-related symptoms.

**Table 2:** Spearman correlation between nutrients and emotional measures.

Micronutrients	Depression (ADI-12)	Anxiety (BAI)
Iodine (μg)	*r* = −0.07 (*p* = 0.570)	*r* = **−0.39** (*p* = 0.002)
Magnesium (mg)	*r* = −0.17 (*p* = 0.188)	*r* = **−0.28** (*p* = 0.031)
Vitamin B6 (mg)	*r* = −0.18 (*p* = 0.175)	*r* = −0.24 (*p* = 0.067)
Vitamin B9 (μg)	*r* = **−0.35** (*p* = 0.006)	*r* = **−0.35** (*p* = 0.006)
Vitamin C (mg)	*r* = −0.22 (*p* = 0.083)	*r* = **−0.28** (*p* = 0.027)
Depression (ADI-12)	--	*r* = **0.52** (*p* < 0.001)

Correlations ≥ | ± 0.25| are statistically significant at the 5% level (*p* < 0.05). They appear in bold. The *p*-value for each correlation is given in parentheses.

Furthermore, the correlation between the scores of two emotional symptoms was strong (0.52), indicating that patients with ALS exhibiting higher depressive scores also tend to report elevated anxiety symptoms.

### 3.3 Predictive model

**Figure 1:**
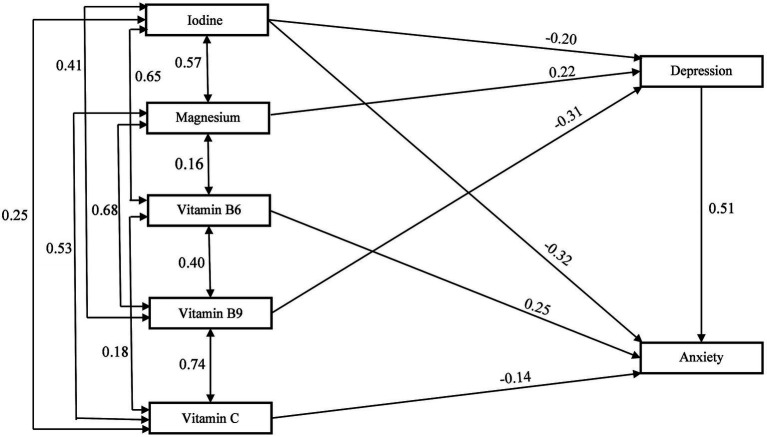
Path analysis model of anxiety-related and depression scores based on micronutrient intake.

A path analysis was conducted in which anxiety-related symptom severity (BAI scores) and depression scores were statistically modeled based on micronutrient intake. The Bollen-Stine bootstrap procedure (35) indicated the presence of multivariate normality (*p* = 0.915); however, the multivariate kurtosis was clearly peaked (Kurtosis = 67.31), so it was decided to estimate the predictive model using unweighted least squares. Figure 1 illustrates the estimated model and standardized regression weights of the nutrients on the scores of both emotional symptoms. While several approximate fit indices suggested a good fit to the data (GFI = 1.000, NFI = 1.000, SRMR = 0.001, and standardized residuals ≥ |2.58| = 0.00%) the *χ^2^/df* ratio was poor (*χ^2^/df* = 29.59); therefore, the model should be interpreted cautiously as exploratory. The only statistic that did not indicate a good fit was *χ^2^/df*, which is not unusual, since in non-multivariate normality tests using the unweighted least squares estimation method, the *χ^2^* value tends to be high (32).

One of the problems with the unweighted least squares method is that it does not estimate the *p*-values of the statistics it calculates. Therefore, it is necessary to use bootstrapping procedures to estimate population parameters and their significance at the population level (32). Furthermore, the bootstrapping procedure is recommended for small samples to estimate population parameters and compare them with sample statistics in order to determine (32): the degree to which results can be generalized to the population, the bias between statistics and parameters, and the significance of parameters in unweighted least squares procedures. We applied this technique using 500 replicate samples of size n = 61 with replacement. In this way, we were able to estimate 500 regression weights and correlations across the resamples, obtaining the mean and associated bias for each parameter. Table 3 shows the results for the regression weights, while Table 4 shows the results for the correlations.

**Table 3:** Bootstrap estimates for regression weights.

Regression path	Regression weights in the sample	Mean of estimated parameters	Bias of estimated parameters (Standard error)	Confidence interval	*p* of the confidence interval
Iodine ➔ Depression	−0.21	−0.17	0.03 (0.01)	(−0.51, 0.17)	0.205
Magnesium ➔ Depression	0.22	0.22	0.00 (0.01)	(−0.25, 0.60)	0.263
B6 ➔ Depression	0.00	0.00	0.00 (0.00)	(0.00, 0.00)	1.000
B9 ➔ Depression	−0.32	−0.33	−0.01 (0.01)	(−0.78, −0.01)	0.045
C ➔ Depression	−0.07	−0.09	−0.02 (0.01)	(−0.36, 0.30)	0.726
Depression ➔ Anxiety	0.51	0.50	0.01 (0.01)	(0.26, 0.69)	0.004
Iodine ➔ Anxiety	−0.31	−0.36	−0.05 (0.01)	(−0.86, 0.07)	0.098
Magnesium ➔ Anxiety	0.09	0.03	0.06 (0.01)	(−0.25, 0.68)	0.480
B6 ➔ Anxiety	0.24	0.29	0.05 (0.01)	(−0.35, 0.65)	0.506
B9 ➔ Anxiety	−0.08	−0.07	0.01 (0.01)	(−0.66, 0.28)	0.637
C ➔ Anxiety	−0.15	−0.17	−0.02 (0.01)	(−0.39, 0.26)	0.451

**Table 4:** Bootstrap estimates for correlations.

Variable pair	Correlations in the sample	Mean of estimated parameters	Bias of estimated parameters (Standard error)	Confidence interval	*p* of the confidence interval
Magnesium and B6	0.16	0.20	0.04 (0.01)	(−0.41, 0.64)	0.852
B6 and Iodine	0.65	0.63	−0.02 (0.01)	(0.26, 0.93)	0.004
Magnesium and Iodine	0.57	0.54	−0.03 (0.01)	(−0.16, 0.81)	0.129
B9 and C	0.74	0.74	0.00 (0.00)	(0.54, 0.86)	0.005
B6 and C	0.18	0.22	0.04 (0.01)	(−0,35, 0.61)	0.901
Magnesium and C	0.53	0.53	0.00 (0.00)	(0.35, 0.70)	0.005
Iodine and C	0.25	0.25	0.00 (0.00)	(−0.13, 0.61)	0.203
B6 and B9	0.40	0.42	0.02 (0.01)	(−0.01, 0.71)	0.096
Magnesium and B9	0.68	0.68	0.00 (0.00)	(0.49, 0.82)	0.005
Iodine and B9	0.41	0.40	−0.01 (0.01)	(0.13, 0.74)	0.003

If we examine the results in Table 3, we can see that the mean of the 500 estimated regression weight parameters exhibits low bias (between −0.05 and 0.06) when compared to the regression weight values of the model in Figure 1, with a very low standard error of bias (between 0.00 and 0.01). A similar pattern can be observed in Table 4 between the model’s correlation values and the mean of the corresponding parameter estimates, the bias (between −0.03 and 0.04), and its standard error (between 0.00 and 0.01). This would indicate that the regression weights of the predictive model are quite similar to those of the population parameters. In the same tables, we can observe the confidence intervals of the estimated parameters and their *p*-values. Only some are statistically significant (*p* < 0.05), that is, different from zero. When working with small samples, it is common for significance tests not to allow the null hypothesis of no relationship to be rejected. In such cases, it is recommended to use effect size values to interpret the results (36), since the effect size does not depend on the sample size, unlike p-values.

According to Cohen’s criteria (36), regression weight values of 0.14 indicate a small effect size, whereas values of 0.36 and 0.51 represent medium and large effect sizes, respectively. Considering only regression paths with weights of at least | ± 0.14|, depression-related symptoms showed exploratory associations with iodine (*β* = −0.21) and magnesium (*β* = 0.22) and were statistically associated with vitamin B9 (*β* = −0.32) intake. Together, these predictors accounted for 13% (*R^2^* = 0.13) of the variance in depression scores. A *post hoc* statistical power (1 – *β*) analysis was calculated based on the sample size (*n* = 61), an *R^2^* = 0.13, five predictors, and a significance level (*α*) of 0.05. The resulting statistical power was 0.91, which is substantially higher than the minimum recommended threshold of 0.80 (23), thereby confirming the statistical robustness of our findings. From a clinical standpoint, this exploratory pattern indicates that patients with ALS reporting higher depression scores exhibited lower dietary intake levels of iodine and vitamin B9, along with higher magnesium intake.

On the other hand, anxiety-related symptom severity (BAI scores) showed exploratory associations with iodine (*β* = −0.31), vitamin B6 (*β* = 0.24), and vitamin C (*β* = −0.15) intake, in addition to the concomitant effect of depression scores (*β* = 0.51). Together, these variables explained 41% of the variance in anxiety-related symptom scores (*R^2^* = 0.41). Given the high variability observed for vitamin B6 intake, sensitivity analyses excluding extreme vitamin B6 values were conducted. These analyses yielded substantially similar results and did not materially alter the observed association between vitamin B6 intake and anxiety-related symptom scores, suggesting that the findings were not driven by outlying observations. Similarly, a *post-hoc* statistical power analysis was calculated for *n* = 61, an *R^2^* = 0.41, six predictors, and *α* = 0.05, yielding an optimal statistical power of 0.99. Sensitivity analyses excluding extreme vitamin B6 values yielded similar estimates and did not materially alter the observed associations. This statistical pattern reflects that patients with ALS with higher depression scores exhibited lower intake levels of iodine and vitamin C, along with higher vitamin B6 intake and higher anxiety symptoms.

## 4 Discussion

The close relationship between ALS and anxiety-related and depression symptoms is strongly established. However, studies focusing on the adequate nutritional intake for patients with ALS remain scarce, and a specific nutritional strategy has not yet been established (37). Consequently, the present work aims to identify specific micronutrients associated with anxiety-related and depression scores within a Spanish cohort of patients diagnosed with ALS. In any case, beyond potential biological mechanisms related to micronutrient intake and neurotransmitter systems, the emotional symptoms observed in ALS are likely to arise from a complex and dynamic interaction of biological, psychological, and social factors. Disease-related functional decline, respiratory impairment, dysphagia, and overall symptom burden may directly contribute to psychological distress. In addition, social isolation, caregiver strain, coping strategies, and access to psychosocial or psychotherapeutic support have all been identified as relevant determinants of anxiety-related and depressive symptoms with motor neuron disease (58). Therefore, while nutritional factors may represent one relevant component in this multifactorial context, they should be interpreted within a broader neurobiopsychosocial framework rather than as isolated explanatory determinants of emotional outcomes.

The correlation analysis showed that vitamin B9 intake is negatively associated with depression scores. We further examined these results by utilizing multivariate models, considering the magnitude of the effect size of the regression weights (*β* ≥ 0.14), the model revealed exploratory associations for iodine and magnesium, whereas vitamin B9 (*β* = −0.32) emerged as an inferentially supported predictor (*p* < 0.05) of depression scores, accounting for 13% of their variance. Specifically, patients with higher depression scores consumed lower amounts of iodine and vitamin B9; but higher amounts of magnesium. Notably, the pattern of relevant variables identified in the path analysis does not completely align with the initial bivariate correlations shown in Table 2. As indicated above, according to Spearman rank correlations, it might have been expected that vitamin B6 and vitamin B9 would emerge as the main correlates of depression, however, vitamin B6 did not retain its statistically significant association with depression in the proposed confirmatory model. This outcome can be explained by the inter-correlations that vitamin B6 presents with vitamin B9 (*r* = 0.40), and with iodine (*r* = 0.65; see Figure 1). Since vitamin B9 presents a medium-high correlation with the other two predictors, the variance that vitamin B9 and depression share is already captured by the other variables in the model. This finding suggests that it is more useful to employ multivariate approaches when analyzing a criterion like depression; if only bivariate Spearman correlation results had been analyzed, the importance of these nutrients on the variable would have been interpreted incorrectly. In other words, this type of multivariate path model facilitates the identification of complex relationships among variables, which prevents the overestimation of certain nutritional impacts on depression scores that would occur if confounding shared variances were ignored (23).

Therefore, although previous research primarily points out the importance of vitamin C intake in preventing the appearance of depression (34), principally because this vitamin is essential for the synthesis and regulation of neurotransmitters such as dopamine, norepinephrine, and serotonin (38), our model indicates that other micronutrients showed exploratory associations with depression scores, according to the magnitude of the effect size of the regression weights (*β* ≥ 0.14). Among these, iodine was one of the relevant variables identified in our model, since its most recognized function is its participation in the synthesis of thyroid hormones T4 and T3, which are fundamental for the regulation of metabolism, growth, and neurological development. In adults, hypothyroidism has been associated with cognitive deterioration and decreased general well-being (39), with evidence of a dose-dependent relationship between subclinical hypothyroidism and depression, a mechanism possibly mediated by alterations in neurotransmitters such as serotonin, norepinephrine, and dopamine (40). In this sense, although some studies have not found a direct relationship between thyroid function and ALS (41), others associate low thyroid hormone levels with neurodegenerative diseases such as Alzheimer’s (42).

Another micronutrient that is negatively associated with depression scores is vitamin B9, supported in this case both by statistical significance (*p* < 0.05) and effect size (*β* ≥ 0.14). These findings are consistent with previous works indicating a protective effect of this vitamin against depression (43, 44), which could be attributed to its importance in the correct functioning of serotonergic and dopaminergic pathways involved in mood regulation (18).

Finally, magnesium also emerged as relevant variable related to depression within our sample. Notably, it exhibited a positive exploratory association, considering the magnitude of the effect size of the regression weights (*β* ≥ 0.14), indicating that a higher magnesium intake is associated with higher depression scores. This finding contrasts with the widely accepted premise regarding the importance of adequate magnesium consumption for maintaining a healthy mood and preventing the appearance of depression (45). However, while low magnesium levels have been traditionally linked to depressive symptoms due to their role in neurotransmission (46), the association is not universally inverse across all population groups. Some research among older adults demonstrates a higher risk of depression associated with very high magnesium intakes, failing to show a clear dose–response protective relationship at elevated levels. Specifically, among individuals over 60 years old, an increase in dietary magnesium intake has been linked to a higher risk of depression (47). This highlights the crucial effect of age on the association between magnesium consumption and depression risk, especially because its metabolic function and mineral absorption capacity undergo changes during aging (48–50). In our study, the mean age of the sample population was 56.43 ± 10.17 years, indicating a cohort close to or exceeding the 60-year threshold. Therefore, nutritional recommendations, particularly regarding magnesium, should be adjusted based on age (51).

As for what we observed in our model regarding anxiety-related symptom severity, before discussing these findings, it is important to acknowledge that the BAI includes several somatic items that may overlap with ALS manifestations, including respiratory difficulties, fatigue, and other physical symptoms. Consequently, BAI scores in populations with ALS may reflect a combination of affective distress, disease-related symptom burden, and heightened awareness of bodily sensations. Therefore, the following interpretations should be understood as referring to anxiety-related symptom burden as measured by the BAI rather than to psychological anxiety as an isolated construct. Regarding BAI-assessed anxiety-related symptoms, our findings indicate that the intake of certain micronutrients correlates significantly with BAI scores in patients with ALS. Specifically, iodine, magnesium, vitamin B9, and vitamin C are negatively related to the BAI scores (Table 2).

However, when establishing multivariate models, certain micronutrients that displayed significant bivariate correlations, specifically magnesium and vitamin B9, did not retain statistical significance for anxiety-related symptom scores. Again, this outcome can be attributed to the substantial inter-correlations observed between vitamin B9 and the remaining predictors that did achieve statistical significance within the model: vitamin C (*r* = 0.74), vitamin B6 (*r* = 0.40), and iodine (*r* = 0.41). A similar pattern occurs with magnesium, which correlated noticeably with iodine (*r* = 0.57) and vitamin C (*r* = 0.53). Therefore, the shared variance of vitamin B9 and magnesium with the other predictors accounts for their lack of independent explanatory power within the multivariate model for anxiety-related symptoms.

B vitamins play a fundamental role for the correct functioning of the brain and nervous system, including cellular processes that help maintain an adequate balance between neural inhibition and excitation. This role is key, because a balance too inclined toward excitation can promote neuropsychiatric disorders such as anxiety (52). This fact could explain how in our multivariate model, higher vitamin B6 consumption was associated with higher the BAI scores observed in patients, contrary to what has been previously published (53), considering that the mean intake we recorded of vitamin B6 is extraordinarily high (with an average consumption of 13.6 mg, when normal intake is 1.7 mg for men and 1.5 mg for women) (54, 55). However, regarding the interpretation of these results, extreme caution is warranted, as relatively high mean and variability observed for vitamin B6 intake may be influenced by population-specific dietary patterns, extreme values, or dietary reporting variability. Although no data-entry errors were identified and sensitivity analyses confirmed the robustness of this association against outliers, the possibility of unreported nutritional practices influencing this exceptional estimate cannot be entirely excluded, necessitating further investigation in larger cohorts.

Conversely, vitamin C showed a negative exploratory association with BAI scores in our study, considering the magnitude of the effect size of the regression weights (*β* ≥ 0.14), reinforcing its possible association with lower anxiety-related symptom burden (56). Furthermore, despite the fact that patients with less anxiety also reported lower depression scores (Table 2; Figure 1), it should be noted that, as indicated above, the lack of vitamin C did not emerge as a significant correlate for depression scores.

Finally, iodine, which is fundamental for monoaminergic neurotransmission (20), exhibited a similar negative exploratory association with BAI-assessed anxiety-related symptom scores, a finding consistent with observations by other authors (57). These findings suggest that iodine intake may be associated with emotional symptom burden in patients with ALS.

In conclusion, our findings suggest that specific patterns of micronutrient intake show exploratory associations with depressive symptoms and self-reported anxiety-related symptoms in patients with ALS, considering an effect size of *β* ≥ 0.14. Nutritional factors may represent one of multiple contributing domains involved in emotional symptom burden in ALS, rather than a primary explanatory determinant within a broader biopsychosocial framework in which functional, psychological, and social determinants play a central role. The cross-sectional design precludes causal inference, and therefore the observed associations should be interpreted as correlational. These associations may also reflect underlying clinical severity, functional status, or broader care-related factors rather than direct effects of specific micronutrient intake on emotional symptoms. Future longitudinal and interventional studies are needed to clarify the directionality and potential clinical relevance of these relationships.

Furthermore, residual confounding and measurement limitations should be explicitly considered when interpreting the present findings. The BAI includes several somatic items that may overlap with ALS-related symptomatology, potentially inflating anxiety-related scores and complicating the distinction between affective distress and disease burden. In addition, several clinically relevant variables were not available in the present study, including disease severity, functional impairment, dysphagia, appetite loss, weight loss or malnutrition, feeding support, psychotropic medication use, ALS subtype, disease duration, socioeconomic status, and psychiatric history. These factors may influence both dietary intake and emotional symptom burden and therefore may partially account for the observed associations between micronutrient intake and anxiety-related and depression scores. Future studies incorporating these variables and employing complementary assessment approaches are warranted to improve interpretability and reduce residual confounding.

In addition, the observed micronutrient patterns are likely to reflect overall dietary intake rather than isolated nutrient effects. In patients with ALS, dietary intake may be influenced by dysphagia, fatigue, reduced appetite, disease burden, and socioeconomic constraints, which should be considered when interpreting nutrient-emotion associations. Consequently, caution is warranted when extrapolating these findings to specific nutritional recommendations or supplementation strategies, as the observed associations may reflect broader dietary and clinical contexts rather than isolated micronutrient effects.

## Data Availability

The raw data supporting the conclusions of this article will be made available by the authors, without undue reservation.

## Footnotes

1
https://dietopro.com/
